# Serum Metabolomic Analysis Suggests Impairment of Myocardial Energy Production in Takotsubo Syndrome

**DOI:** 10.3390/metabo11070439

**Published:** 2021-07-03

**Authors:** Iván J. Nuñez-Gil, Mireia Andrés, Begoña Benito, Esther Bernardo, Oscar Vedia, Ignacio Ferreira-Gonzalez, Ignasi Barba

**Affiliations:** 1 Interventional Cardiology Unit, Cardiovascular Institute, Hospital Clínico San Carlos, Calle del Prof Martín Lagos, s/n, 28040 Madrid, Spain; ivanjavier.nunez@salud.madrid.org (I.J.N.-G.); esther.bernardo@salud.madrid.org (E.B.); oscar.vedia@salud.madrid.org (O.V.); 2 Cardiovascular Diseases Research Group, Department of Cardiology, Vall d’Hebron Institut de Recerca (VHIR), Vall d’Hebron Hospital Universitari, Passeig Vall d’Hebron 119-129, 08035 Barcelona, Spain; mireia.andres@vhir.org (M.A.); bergona.benito@vhir.org (B.B.); 3 Centro de Investigación Biomédica en Red (CIBER) de Enfermedades Cardiovasculares (CIBERCV), Instituto de Salud Carlos III, 28029 Madrid, Spain; 4 Faculty of Medicine, University of Vic-Central University of Catalonia (UVicUCC), Can Baumann, Ctra, de Roda, 70, 08500 Vic, Spain; 5 Vall d’Hebron Institut d’Oncologia (VHIO), CELLEX CENTER C/ Natzaret 115-117, 08035 Barcelona, Spain

**Keywords:** metabolomics, ^1^H NMR spectroscopy, acute coronary syndromes, takotsubo syndrome, apical ballooning

## Abstract

Introduction: Takotsubo syndrome is a complex entity that, although it usually has a good prognosis, can be life threatening. While recent advances have improved the knowledge of takotsubo syndrome, many aspects of its etiology still remain uncertain. Metabolomics, a hypothesis generating approach, could provide novel pathophysiology information about this disease. Methods and Results: Serum samples were obtained from takotsubo (*n* = 19) and acute myocardial infarction patients (*n* = 8) at the cath lab and, in the case of takotsubo, again once the patient had recovered, 3 months after the main event. ^1^H NMR spectra of the serum were acquired at 9.4T using a CPMG pulse sequence (32 ms effective delay). Supervised and unsupervised pattern recognition approaches where applied to the data. Pattern recognition was able to differentiate between takotsubo and acute myocardial infarction during the acute phase with 95% accuracy. Myocardial infarction patients showed an increase in lipid signals, a known risk factor for the disease while takotsubo patients showed a relative increase in acetate that could suggest a reduced turnover of the Krebs cycle. When comparing acute and recovered phases, we could detect an increase in alanine and creatine once patients recovered. Conclusions: Our results demonstrate that takotsubo syndrome is metabolically different than AMI, showing limited myocardial energy production capacity during the acute phase. We achieved high classification success against AMI; however, this study should be considered as a proof of concept regarding clinical application of metabolic profiling in takotsubo cardiomyopathy.

## 1. Introduction

Takotsubo syndrome (TKS), also called apical ballooning syndrome, is a non-ischemic cardiomyopathy characterized by transient apical dyskinesia. Clinical presentation usually mimics an acute myocardial infarction (AMI), including chest pain, electrocardiogram (EKG) changes (frequently ST elevation), and apical dyskinesia, but without coronary occlusion or significant coronary disease as the underlying mechanism of the acute event [1]. The most frequent and first described pattern is dyskinesia of the apex, or apical and mid segments of the left ventricle (LV) [2]; however, atypical TKS with mid-ventricular dyskinesia has also been reported [3]. EKG changes as well as contractility abnormalities tend to recover in days or weeks. However, despite contractility disturbances being reversible and the recovery of ejection fraction usually complete, TKS is far from being benign, potentially leading to heart failure, mainly in patients with other comorbidities and poor previous functional class [4,5].

TKS syndrome is usually associated with a triggering stressful event, either physical [6] or involving emotional trauma [7]. It has been reported that the type of trigger may have prognostic relevance [8]. It is believed that an intense release of catecholamines could drive the initial trigger of the TKS, although the mechanisms explaining such association are not yet clear [9].

The metabolome is the set of metabolites found in an organism, tissue, or biofluid. Unlike the genome, the metabolome is affected by environmental factors, including disease status; thus, it has potential clinical applicability as a diagnostic tool [10]. The cardiovascular field has been at the forefront of clinical metabolomic applications in biomarker discovery, and also as a holistic approach to understand complex disorders [11] including cardiovascular diseases [12,13,14,15]. In this sense, metabolomics has proven power to assist our understanding of the metabolic derangements associated to myocardial dysfunction [16] and heart failure [14,17,18,19]. However, to our knowledge, there are no metabolomic studies of TKS.

The etiology of TKS is not yet fully understood; in this context, a hypothesis-generating approach such as ^1^H NMR metabolic fingerprinting could provide information regarding the pathophysiology of this disorder, with the added benefit of detecting potential novel biomarkers on top of the previously known risk factors. The main objective of the present work was, therefore, to gain insight of TKS pathophysiology using ^1^H NMR based metabolomics.

## 2. Results

### 2.1. Study Population

Clinical characteristics of the study population are shown in Table 1. Among TKS patients, mean age was 71 years, and, as expected for a TKS population, a great majority (19/20, 95%) were women. The prevalence of cardiovascular risk factors was hypertension 45%, dyslipidemia 50%, diabetes 30%, smokers 30%, and family history 55%. Four patients received antiplatelet agents, and two others were on oral anticoagulants prior to the index episode. Presentation was as an AMI with ST elevation in all cases. All but 2 patients were treated with aspirin, and 10 patients with dual antiplatelet therapy. Betablockers, angiotensin-converting enzyme inhibitors or angiotensin receptor blockers, and statins were given to 76%, 72%, and 72% of patients, respectively.

### 2.2. Visual Inspection

**Figure 1:**
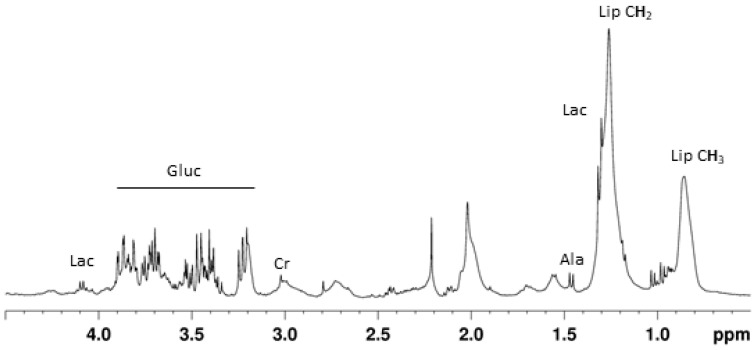
Typical ^1^H NMR spectra of serum from the acute phase of TKS. The spectrum was acquired using a CPMG pulse sequence with an effective T2 delay of 32 ms. Lac—lactate; Gluc—glucose; Cr—creatine, Ala—alanine; Lip CH_2_—lipid methylene moieties; and Lip CH_3_—lipid methyl moieties.

For the whole TKS population, NMR spectra obtained in the present work were similar to previously published data by ourselves [20,21] and others [22]. Figure 1 shows a representative spectrum dominated by the peaks corresponding to lipid moieties, lactate, and glucose. Other identified metabolites were amino acids (Leucine, Isoleucine, Valine), acetate, and creatine. On visual inspection alone, we could not find conspicuous differences between the spectra of TKS and AMI patients.

### 2.3. Pattern Recognition

#### 2.3.1. The Acute Phase: Takotsubo vs. AMI

**Figure 2:**
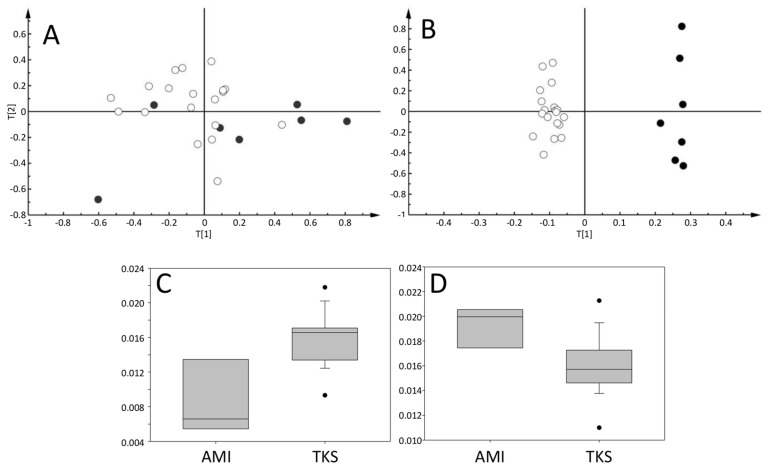
Pattern recognition analysis in the acute phase of TKS (*n* = 19) (white circles), and AMI patients (*n* = 8) (black dots). (**A**) corresponds to the score plot of the principal component analysis, showing a tendency towards classification. (**B**) is the score plot of the OPLS-DA of the same samples. (**C**,**D**) correspond to box plots for acetate (1.93 ppm) and methylene lipid peaks (1.28 ppm), respectively, which were identified as being responsible for the differences in the OPLS-DA analysis.

When comparing TKS and AMI samples obtained during the acute phase, principal component analysis (PCA) showed a tendency towards grouping the samples according to pathology (Figure 2A). Principal component analysis, a form of unsupervised classification, organizes the samples according to the sources of variation in orthogonal vectors in a descending manner. The grouping observed is completely unbiased and shows that the main sources of variation in the metabolic profiles are associated with the disease (component 1, horizontal axis in Figure 2A) although classification success is not very high.

On the other hand, using supervised classification that focuses on the differences between two pre-set groups of samples, it is possible to obtain a statistically significant orthogonal projection to a latent structures-discriminant analysis (OPLS-DA) model (*p* < 0.01) able to correctly classify 96% (26/27) of the samples (19 with TKS and 9 with AMI, Figure 2B). The metabolites responsible for these differences were elevated lipids (*p* = 0.02) in AMI patients and elevated acetate (*p* < 0.01) in TKS patients (Figure 2C,D, respectively).

#### 2.3.2. Evolution of Takotsubo Metabolic Profile: Acute vs. Subacute Phases

**Figure 3:**
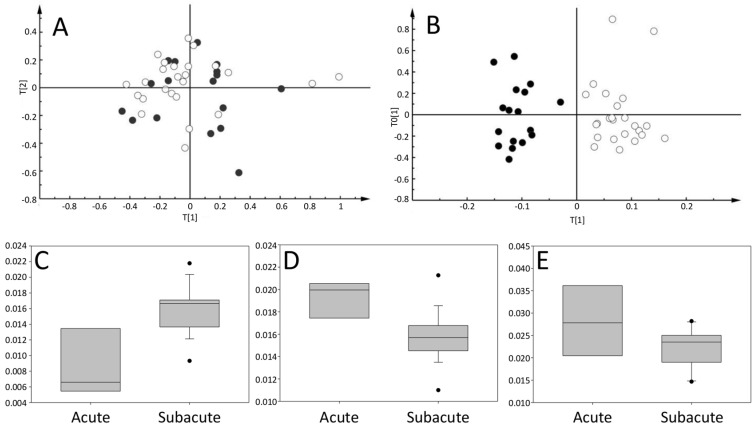
Classification between acute and subacute phase of TKS. (**A**) score plot of the principal component analysis. (**B**) OPLS-DA score plot of the same samples as shown in A; black dots correspond to acute (*n* = 19) and white circles to subacute samples (*n* = 28). (**C**–**E**) correspond to the box plots for Alanine, Creatine, and Glucose, respectively, between acute and subacute TKS samples.

The principal component analysis did not show clear differences between samples from TKS patients obtained in the acute (*n* = 19) and subacute (*n* = 28) phases. The first components, which explain the highest variance in the dataset, are not related to sample grouping according to the pathology (Figure 3A).

On the other hand, supervised classification using OPLS-DA was able to classify 89% of the TKS samples between acute and subacute (Figure 3B). The analysis showed that the most relevant variables for the classification were the peaks at 3.87, 1.47, and 3.03 ppm, which were tentatively assigned to glucose, creatine, and alanine based on 2D NMR spectroscopy and comparison to public databases [23].

When focusing on those variables, alanine and creatine were found to be increased and reduced, respectively, during the acute phase compared with the subacute phase (*p* = 0.008 and *p* = 0.007, respectively), while glucose was not statistically different (Figure 3C–E).

## 3. Discussion

In this work, we identified the particular ^1^H NMR-based metabolic fingerprinting of serum samples from TKS patients, providing novel information on the differentiation between this disorder and the AMI in the acute phase, and describing the temporal evolution of the metabolic pattern in the acute and subacute phases, giving new insights into the pathophysiology of the TKS.

TKS patients included in the study were mostly females (96%) with a mean age of 71 ± 10 years. When angiography was performed during the event no significant coronary occlusion or significant lesions in the arteries could be seen. Imaging techniques showed dyskinesia of the apex, apical, and mid segments of the LV. In addition, the trigger of the event was usually associated to emotional stress. These clinical and epidemiological characteristics are typical of TKS syndrome and are similar to other series previously published from our environment [24,25] including reduced creatine kinase or troponin elevation in the case of TKS compared with AMI patients. Our findings are in agreement with the seminal paper by Wittstein et al. [7] that only found mildly elevated myocardial necrosis markers (i.e., troponin) in the TKS group.

### 3.1. The Acute Phase: Takotsubo vs. AMI 

The differences observed between TKS and acute myocardial infarction clearly point to different metabolic pathophysiology backgrounds in the two pathologies.

TKS patients have higher levels of acetate and, in the heart, acetate is mainly metabolized in the Krebs cycle [26]. An accumulation of acetate may be associated with reduced activity in the Krebs cycle, leading to a reduced energy production and subsequent myocardial stunning seen in those patients. On the other hand, AMI patients showed higher levels of lipids, being consistent with dyslipidemia as a risk factor for the disease [27]. The increase in circulating myocardial necrosis biomarkers is associated to membrane rupture that would also allow intracellular metabolites to reach circulation. Although it cannot be discarded that membrane rupture plays a role in the metabolic differences between AMI and TKS, the fact that some metabolites increase while others decrease makes it unlikely that this is the main reason for the differences observed.

At presentation, acute myocardial infarction and TKS syndrome showed similar symptoms; differential diagnosis cannot be fully established without invasive coronarography and evolution. In this work, we provide preliminary evidence showing that the serum metabolic profiling could be used as a diagnostic tool.

### 3.2. Differences between Acute and Subacute Phases

Pattern recognition is able to differentiate the metabolic pattern obtained during the acute phase of TKS from the recovered phase. By comparing the same population during the acute phase and after recovery, we are able to minimize the effects on the metabolic profile associated to differences in the population, such as gender [28,29] and age [30].

Reduced alanine levels in the acute phase would suggest a reduction in anaerobic glycolysis. We could not detect differences in the amount of lactate, another marker of anaerobic glycolysis between AMI and acute phase TKS, which could be due to the high, broad, lipid peaks in AMI masking differences in peaks that appear close in the spectra.

When taken together, our data suggests that acute TKS syndrome is associated to a reduced energy production both at the mitochondrial level and glycolysis. Low energy availability would be consistent with apical dyskinesia typical of TKS syndrome. Furthermore, TKS syndrome has been associated with an impaired energy metabolism [31] that can last over time [32] as shown by ^31^P NMR spectroscopy; however, these studies compare TKS patients with controls and not AMI patients, as in our study. At the present time, our results do not allow us to speculate about the mechanisms leading to the metabolic impairment.

Our results of classification success rates of nearly 90% with (95% confidence interval) are slightly better than for coronary artery disease of between 80 and 60% classification rate with 99% confidence interval [28]. The increase in classification success compared with coronary artery disease may be due to the fact that TKS is a rare disease that affects a relatively homogeneous population and has less variation than coronary artery disease which affects a great variety of patients and has different presentations. Furthermore, our proof-of-concept study incorporates a relatively low number of patients; further studies with larger cohorts of patients should evaluate the accuracy of metabolic profiling as a diagnostic tool. Larger studies would also provide information about the prognostic value of metabolomic profiling as the analysis of current data looks promising but did not reach statistical significance (Figure S1).

Metabolomics is fast and cost effective on a per-sample basis [33] thus it is ideally suited for the clinical environment. However, it has not reached everyday clinical practice. The opening of large phenome centers may bridge the gap between clinical research and being able to influence clinical decision-making in the hospital environment for both medical and surgical treatments [34]. Knowledge regarding non-common diseases such as TKS cardiomyopathy would facilitate the clinical implementation of metabolomics to the clinic once the last technical hurdles are solved.

Differential diagnosis between TKS and acute myocardial infarction is usually challenging and based on transient ventricular motion abnormalities with the absence of coronary lesions potentially responsible. Moreover, electrocardiographic data is not always able to differentiate between TKS cardiomyopathy and AMI [35,36]. On the other hand, the dynamics and peak levels of cardiac-specific troponin-I (cTn), the biomarker base of the diagnosis in both entities, could orientate us but is not definitive either. Usually, in TKS the magnitude of increase in the biomarkers is less than that observed with an AMI and disproportionately low regarding the marked an extensive acute regional wall motion abnormality displayed in most TKS. Interestingly, biomarker elevation and left ventricular abnormalities resolve before the electrocardiogram. However, these special features are not useful to establish a priori diagnostic differences.

Our data suggest that the metabolic phenotype of TKS syndrome is different from acute myocardial infarction further supporting the notion of TKS being a different physiological entity.

Study limitations: The drawback of performing non-invasive studies in human is that we assume that the metabolic fingerprint of the heart can be detected on the systemic circulation. However, by using AMI controls during the acute phase and also TKS patients once they have recovered, we minimize the possibilities of effects other than cardiac. Furthermore, the presence of lipoproteins that may originate in the diet have a direct inference on myocardial lipid metabolism (β-oxidation) using this simple approach.

Another limitation is that the number of patients included is relatively low, especially when deriving conclusions aimed at possible clinical applications. The low numbers precluded us to analyze the relevance of some important clinical features (type of diabetes, renal function, number of diseased vessels, etc.). Thus, we considered both groups aggregated in a pragmatic way. In this regard, we consider the present study as a “proof of concept” and that prospective studies with larger cohorts of patients should be done prior to clinical application.

In conclusion, metabolic profiling suggests limited myocardial energy production capacity in the acute phase of TKS cardiomyopathy. Furthermore, the high classification success between AMI and TKS achieved in this study should be considered as a proof of concept to be further investigated.

## 4. Material and Methods

### 4.1. Patients

A total of 25 patients with TKS were included in the study, from whom 47 valid samples were obtained: 19 at the cath lab during coronary angiography in the acute phase, and 28 during a follow up visit 3 months after the event. Additionally, 8 samples were obtained from an independent cohort of 8 patients with AMI at the cath lab during angioplasty procedure, who served as controls. All samples came from the PLATAKO study [25]. The protocol was approved by the ethics committee of the Hospital Clínico San Carlos; all patients provided written consent to participate in the study.

Blood samples were collected in serum gel tubes (Vacutainer), allowed to clot for 30 min, and then centrifuged for 5 min at 1000 g 4 °C. Serum samples were stored at −80 °C until use.

### 4.2. NMR Spectroscopy

For NMR spectroscopy, 200 μL of serum was mixed with 300 μL of deuterium oxide and transferred to a 5 mm NMR tube. Spectra were obtained at 30C on a Bruker Avance 400 spectrometer (Bruker, Madrid, Spain) interfaced to a 9.4 T vertical bore magnet using a CPMG pulse sequence with an effective T2 delay of 32 milliseconds. Each spectrum consisted in the accumulation of 64 scans with a total acquisition time of approximately 6 min [21,37]. For metabolite identification purposes, ^1^H-^1^H TOCSY and ^1^H-^13^C HSQC spectra were acquired from selected samples.

### 4.3. Pattern Recognition

Pattern recognition was done as previously described [20,38]. Briefly, each spectrum was manually phase corrected and divided into 1000 bins of equal width of 0.01 ppm. The resulting digitized spectra was normalized to total area of 1 and fed into SIMCA software (MKS Data Analytics Solutions, Umetrics, Umea, Sweden) for further processing.

General variation within the dataset was analyzed using PCA. This process allowed the variation in the dataset to be organized in orthogonal vectors, each explaining one source of variation in a descending manner. Reducing the number of variables from the dataset facilitated the extraction of information, allowing identification of the main sources of variation within a dataset. PCA is an “unsupervised” approach, meaning that it does not require input from the observer and, thus, is free from possible bias.

In order to assess the capacity of the NMR spectra to differentiate between groups of samples, we used OPLS-DA implemented in SIMCA. This supervised approach aims to develop a statistical model able to differentiate two (or more) populations defined in advance. The group information required for this classification was provided from a “training set” of correctly classified individuals and the classification models were evaluated using the “leave-one-out” approach, where a certain number of samples within the dataset were not used to define the model and later used to test the classification algorithm; this process is repeated in an iterative process until all the samples have gone through both the training and test sets. The evaluation of supervised classification methods is critical to avoid obtaining spurious models; in this work statistical significance of the OPLS-DA was evaluated using the cross-validated predictive residuals, CV-ANOVA, and considered significant when *p* < 0.05 [39].

### 4.4. Statistics

Differences between groups regarding quantitative clinical variables were assessed using one way ANOVA. Frequencies were compared using the chi-square test. Analyses were performed using R-software (http://www.R-project.org, accessed on 29 April 2021) Data are expressed as median ± standard deviation. Differences were considered significant when a two-tailed *p* < 0.05 was found.

## Figures and Tables

**Table 1:** Clinical and epidemiological characteristics of the patients included in this study.

	Cases	Controls
Age (years)	71.54 ± 10.41	67.75 ± 17.39
Hypertension	45%	75%
Dyslipidemia	50%	75%
Diabetes	30%	25%
Smoker	30%	50%
Family History	55%	25%
Obesity	4%	50%
ASA	89%	0%
Statins	72%	0%
Anxiolytics	4%	0%
Antidepressant	8%	0%

## Data Availability

Data will be provided on reasonable request. The data are not publicly available due to privacy restrictions.

## Supplementary Materials

The following are available online at https://www.mdpi.com/article/10.3390/metabo11070439/s1, Figure S1: This figure shows the OPLS-DA model obtained when trying to predict outcome of TKS patients using the simples obtained during the acute phase.
